# Effects of Phthalate Mixtures on Ovarian Folliculogenesis and Steroidogenesis

**DOI:** 10.3390/toxics10050251

**Published:** 2022-05-16

**Authors:** Endia J. Fletcher, Ramsés Santacruz-Márquez, Vasiliki E. Mourikes, Alison M. Neff, Mary J. Laws, Jodi A. Flaws

**Affiliations:** Department of Comparative Biosciences, University of Illinois at Urbana-Champaign, Urbana, IL 61802, USA; endiaf2@illinois.edu (E.J.F.); ramseses@illinois.edu (R.S.-M.); mourike2@illinois.edu (V.E.M.); hantak@illinois.edu (A.M.N.); marylaws@illinois.edu (M.J.L.)

**Keywords:** phthalates, phthalate mixtures, ovary, female reproduction, prenatal exposure

## Abstract

The female reproductive system is dependent upon the health of the ovaries. The ovaries are responsible for regulating reproduction and endocrine function. Throughout a female’s reproductive lifespan, the ovaries undergo continual structural changes that are crucial for the maturation of ovarian follicles and the production of sex steroid hormones. Phthalates are known to target the ovaries at critical time points and to disrupt normal reproductive function. The US population is constantly exposed to measurable levels of phthalates. Phthalates can also pass placental barriers and affect the developing offspring. Phthalates are frequently prevalent as mixtures; however, most previous studies have focused on the effects of single phthalates on the ovary and female reproduction. Thus, the effects of exposure to phthalate mixtures on ovarian function and the female reproductive system remain unclear. Following a brief introduction to the ovary and its major roles, this review covers what is currently known about the effects of phthalate mixtures on the ovary, focusing primarily on their effects on folliculogenesis and steroidogenesis. Furthermore, this review focuses on the effects of phthalate mixtures on female reproductive outcomes. Finally, this review emphasizes the need for future research on the effects of environmentally relevant phthalate mixtures on the ovary and female reproduction.

## 1. Introduction

Endocrine-disrupting chemicals (EDCs) are defined as substances that interfere with the normal synthesis, secretion, binding, and/or action of hormones. EDCs can be found in the environment, food, and consumer products. EDCs can affect both the female and male reproductive systems, but they can also affect a variety of other physiological systems, leading to other underlying health issues such as cancer, obesity, and cardiovascular diseases [1]. EDCs are a public health concern due to their possible long-term effects on human health and reproduction. Phthalates are EDCs of significant importance. Phthalates are a group of chemicals (diesters of phthalic acid) used to make plastics more durable and flexible, and they are used as solvents in various consumer products. Phthalates are a public health concern because most of the US population is known to be subject to measurable phthalate exposure levels [2]. Phthalates are found in shampoo, perfume, food packaging, beverages, household cleaners, shower curtains, building materials, and garden hoses. The Centers for Disease Control and Prevention (CDC), in the Fourth National Report on Human Exposure to Environmental Chemicals, found 13 phthalate metabolites in human blood, serum, and urine samples [2]. Since more than one phthalate was found in each of the collected samples, it is important to note that phthalates are frequently prevalent in the environment as mixtures. Understanding the reproductive effects of phthalate mixtures that are environmentally relevant and mimic human exposure is imperative for developing strategies to protect reproductive health.

The ovary is a key female reproductive organ that is essential for fertility and normal hormone production. It is also a known target of phthalates. This systematic review focuses on integrating data from previous studies to further understand the mechanisms by which phthalates disrupt ovarian function. The literature reviewed in this manuscript was selected to highlight the major impacts phthalates can have on ovarian function (folliculogenesis and steroidogenesis) and reproductive processes regulated by ovarian function. This review also includes mention of some single phthalate studies if the single phthalate studies focused on single phthalates present in the mixtures described in this review, focused on the ovary, and measured the same reproductive outcomes as the mixture studies. This review also highlights the gaps within the current literature and ultimately emphasizes the need for more research on understanding the effects of both single phthalates and phthalate mixtures on the ovary and female fertility.

## 2. Methods

A PubMed search was conducted to identify relevant studies on the effects of phthalate mixtures on female reproduction. Topics of interest included those related to ovarian function, with an emphasis on exposure to phthalate mixtures impacting the ovaries and major processes regulated by the ovary. Keywords searched were phthalates, prenatal exposure, in utero exposure, female reproduction, folliculogenesis, steroidogenesis, and phthalate mixtures. Using the defined criteria, a broad inventory of studies was further refined and selected based on similarity in reproductive outcomes assessed in the studies and relevance to known papers focusing on exposure to phthalate mixtures. In some cases, studies on single phthalates were included if they focused on a single phthalate present in the described mixtures and focused on ovarian function. The evidence was then synthesized by exposure and summarized.

## 3. Development of the Ovary

**Figure 1:**
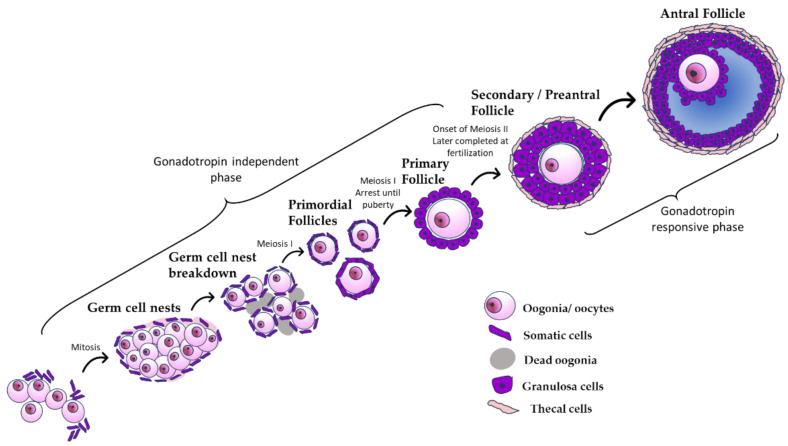
The process of folliculogenesis. The schematic shows that primordial germ cells form germ cell nests, which subsequently break down to form a finite pool of primordial follicles, starting the process of folliculogenesis. During folliculogenesis, primordial follicles grow and mature into primary follicles, then preantral follicles, and finally antral follicles.

The female reproductive system is dependent on the development and health of the ovary. The ovary is a complex organ that undergoes continual structural and functional changes throughout a female’s reproductive lifespan [3]. The main functions of the ovary are to produce oocytes and to synthesize and secrete the hormones that are necessary for reproductive function. The initial fate of the ovary is set during embryonic development when primordial germ cells migrate to the developing ovary, forming germ cell nests [4]. During embryonic development, the germ cell nests are broken down and subsequently assemble with somatic cells to form a finite pool of primordial follicles, starting the process of folliculogenesis (Figure 1). During folliculogenesis, primordial follicles grow and mature into primary follicles, then preantral follicles, and finally antral follicles (Figure 1). Antral follicles are the only follicles that are capable of ovulation and the production of large amounts of sex steroid hormones. Only about 1% of antral follicles will go on to ovulate; about 99% will undergo natural follicular death by atresia [5].

Before puberty, the oocytes in primordial follicles are arrested in prophase I of meiosis. During puberty, gonadotropin-releasing hormone (GnRH) is released from the hypothalamus. This signals the anterior pituitary to secrete gonadotropic hormones known as follicle-stimulating hormone (FSH) and luteinizing hormone (LH) [6]. These hormones help stimulate follicular growth and cause the primordial oocytes to complete meiosis I, forming secondary oocytes. Lastly, under the influence of hormones, the follicle develops into a mature antral follicle with a fluid-filled cavity surrounding the oocyte called an antrum (Figure 1). After the oocyte is fully matured, it can be released from the antral follicle during ovulation into the oviduct, where it can be fertilized. After the oocyte is released from the follicle, the remaining cells undergo physical and chemical changes to form the corpus luteum (CL). The primary function of the corpus luteum is to secrete progesterone, which helps the uterus become a healthy environment for pregnancy. If there is no signal of successful fertilization and implantation, the CL regresses, and the process of follicular maturation restarts. Due to the finite number of primordial follicles present at or around the time of birth, the ovary is eventually depleted of follicles, either through ovulation or atresia. This leads to the onset of reproductive senescence or menopause [6].

## 4. Steroidogenesis

Another important function of the ovary is to synthesize and secrete sex steroid hormones [7]. Sex steroid hormones play a vital role in the growth and differentiation of reproductive tissues that regulate fertility [8]. The entire reproductive cycle depends on the ability of somatic cells inside the ovary to secrete sex steroid hormones. Steroidogenesis is a multi-step process [9]. Mature antral follicles and the corpus luteum are the major producers of sex steroid hormones [7]. Antral follicles contain thecal and granulosa cells. Thecal cells respond to LH, whereas granulosa cells respond to FSH. In response to LH, thecal cells take in cholesterol from lipoproteins within the blood vessels of the ovary and transport it to the inner mitochondrial membrane via the steroidogenic protein known as steroidogenic acute regulatory protein (STAR) [10]. Cholesterol is converted into pregnenolone via the cholesterol side-chain cleavage enzyme (CYP11A1). Next, pregnenolone is transported from the inner mitochondrial membrane to the smooth endoplasmic reticulum and converted to dehydroepiandrosterone and progesterone via 3β-hydroxysteroid dehydrogenase (HSD3B) or cytochrome P450 17A1 (CYP17A1) [7]. These same steroidogenic enzymes help convert progesterone into the androgens androstenedione and testosterone. After androstenedione is produced, it can either diffuse to granulosa cells immediately or be converted to testosterone via 17β-hydroxysteroid dehydrogenase (HSD17B) [7]. After androstenedione diffuses to granulosa cells, it is converted into estrone, a weak estrogen, by CYP19A1. This process is regulated by FSH. Testosterone also diffuses from the thecal cells to the granulosa cells, where it can be converted along with estrone into the most potent form of estrogen, estradiol, via CYP19A1 and HSD17B. The process of steroidogenesis regulates follicular growth in the ovary, determines the stage of the reproductive cycle, and maintains and regulates reproductive function.

## 5. Phthalates

Although phthalates are used as plasticizers in polymers, they are not chemically bound to the polymers. This means that phthalates have the potential to leach out of products, especially when phthalate-containing products are exposed to high temperatures [11]. In addition, diester phthalates, common among consumer products, can rapidly enter the body and undergo metabolism. Monoester phthalates are formed when the diester phthalates present in plastics are metabolized by the gut and liver [12]. Some of these metabolites are more toxic than the parent compounds [13]. Based on analysis of urine samples, the average human exposure to phthalate diesters varies among compounds. For example, in humans, diethyl phthalate (DEP) exposure may range from 2.32 to 12  μg/kg/day, butylbenzyl phthalate (BBP) exposure may range from 0.26 to 0.88  μg/kg/day, dibutyl phthalate (DBP) exposure may range from 0.84 to 5.22  μg/kg/day, di-isobutyl phthalate (DiBP) exposure may range from 0.12 to 1.4  μg/kg/day, and di(2-ethylhexyly) phthalate (DEHP) exposure may range from 3 to 30  μg/kg/day in US and German populations [14,15]. In comparison, some phthalate metabolites can range from 23.8 to 1090 ng/mL for monoethyl (MEP), 43 to 437 ng/mL for monobutyl (MBP), 12.4 to 186 ng/mL for monobenzyl (MBzP), and 1.3 to 31.1 ng/mL for mono(2-ethylhexyl) (MEHP) in adult women [16]. Interestingly, women are more likely to be exposed to phthalates than men. This could be linked to the more frequent use of cosmetics and personal care products by women compared to men [17,18,19]. Exposure to phthalates and their metabolites can interfere with ovarian function by targeting both folliculogenesis and steroidogenesis.

Current research has shown that single phthalates are toxic to female reproductive health. Phthalates are known to target the ovary at all stages of development and adulthood, causing premature ovarian failure, anovulation, infertility, and decreased steroidogenesis [7]. Although numerous studies have assessed the effects of single phthalates on female reproduction, single phthalate exposure does not often mimic a real-world environment. Research has shown that phthalates are frequently prevalent as mixtures, and humans encounter multiple phthalates at once [2]. However, few studies have examined the impact of phthalate mixtures on the ovary and female reproductive health. Thus, this review highlights the current information on the effects of phthalate mixtures on ovarian folliculogenesis and steroidogenesis and emphasizes the need for more research regarding exposure to phthalate mixtures and their effects on the ovary and female reproduction.

## 6. Effects of Single Phthalate and Phthalate Mixture Exposure on the Ovary In Vitro

Various primary in vitro systems, including neonatal ovary, antral follicle, granulosa cell, and oocyte cultures have been utilized to uncover the mechanisms underlying phthalate toxicity in vitro. In some experiments, antral follicles were isolated from the adult ovary and exposed to a vehicle control or an environmentally relevant phthalate mixture containing DEP, DBP, DiBP, BBzP, DEHP, and DiNP. The results indicate that the phthalate mixture decreased antral follicle growth, increased oocyte fragmentation, and decreased the levels of secreted steroid hormones including androstenedione, testosterone, estrone, and estradiol compared to the control [20]. Further, in an in vitro system of ovulation, an environmentally relevant phthalate mixture containing DEP, DBP, DiBP, BBzP, DEHP, and DiNP impaired ovulation and decreased progesterone-regulated genes in mouse antral follicles [21].

Although it is not known which of the chemicals in the mixture are responsible for the adverse outcomes, studies on single phthalates indicate that two of the phthalates in the mixture, DEHP and DBP, inhibit antral follicle growth [22,23]. Further, single phthalate studies show that DEHP exposure causes adverse effects in vitro by altering cell cycle regulators and apoptotic factors, inducing oxidative stress and altering the steroidogenic machinery, and that DPB exposure leads to cytotoxicity by increasing cell cycle arrest in antral follicles [20,22,23,24].

Much like phthalate exposure in the antral follicle, phthalate exposure has detrimental effects on the neonatal ovary in vitro. An environmentally relevant diester phthalate mixture containing DEP, DBP, DiBP, BBzP, DEHP, and DiNP increased apoptosis compared to controls in neonatal ovaries [25]. It is likely that DEHP in the mixture contributed to the toxic effects of the mixture on the neonatal ovary. Studies using single phthalates indicated that exposure to DEHP caused DNA damage, increased oxidative stress, delayed progression of meiotic prophase I, and disrupted homologous recombination in an estrogen-receptor-dependent manner compared to controls in neonatal ovary cultures [26]. Further, DEHP exposure alone impaired germ cell nest breakdown and impaired primordial follicle assembly compared to controls in neonatal ovary cultures [27,28,29].

Other studies have examined the effects of a mixture of monoester phthalates on neonatal ovaries. This is because diester phthalates can be metabolized to monoester phthalates by neonatal ovaries [30]. In one study, neonatal ovaries were exposed to a vehicle control or a monoester phthalate mixture containing MEP, MEHP, MBP, monoisononyl phthalate (MNP), monoisobutyl phthalate (MiBP), and MBzP. The results indicated that the monoester phthalate mixture increased apoptosis and the expression of anti-apoptotic factors compared to the control [25]. Interestingly, this same monoester phthalate mixture impaired follicle growth, altered the levels of secreted sex steroids, and altered the expression of steroidogenic enzymes relative to the control in antral follicles [14]. It is likely that the MEHP in the mixture contributed to the toxicity of the mixture in neonatal ovaries and antral follicles. In studies using single phthalates, MEHP impaired follicle growth, inhibited steroidogenesis, induced oxidative stress in antral follicles, and accelerated primordial follicle recruitment in neonatal ovaries [31,32,33]. The MBP in the mixture may not contribute to the toxicity of the mixture; one study showed that MBP did not affect follicle growth compared to the control [23].

Primary cultures of individual ovarian cell types have further elucidated the mechanisms by which phthalates cause toxicity. However, these studies have focused on single phthalates and not mixtures. In some studies, primary cultures of mural granulosa cells from patients undergoing in vitro fertilization were exposed to vehicle control or DBP, and differentially expressed mRNAs were sequenced. DBP exposure decreased the production of estradiol and progesterone and altered the expression of steroidogenic and angiogenic genes compared to controls in human mural granulosa cells [34]. However, MBP, the primary metabolite of DBP, led to an increase in progesterone production compared to controls in mouse primary granulosa cells [35]. In other studies, DEHP exposure induced apoptosis of denuded mouse oocytes in a dose-dependent manner compared to controls [36]. Interestingly, oocyte apoptosis was attenuated when the oocytes were cultured as cumulous–oocyte complexes, highlighting the importance of the granulosa cell in protecting the oocyte from the negative effects of phthalates [36].

Although previous studies on the effects of phthalates on isolated ovaries, follicles, and ovarian cells provide important information on phthalate toxicity, more studies on the effects of phthalate mixtures on isolated follicles, neonatal ovaries, granulosa cells, thecal cells, and oocytes are necessary to fully understand the toxicity of phthalates. Single phthalate chemicals present in phthalate mixtures should also be more closely evaluated to help determine whether specific phthalates in the mixture behave synergistically or independently to cause toxicity.

## 7. Effects of Postnatal Single Phthalate and Phthalate Mixture Exposure on Ovarian Function and Female Reproduction

In vivo exposure to phthalate mixtures has been found to negatively impact female reproduction by targeting the ovaries at critical times of follicular growth and steroidogenesis. A study using SD rats as a model evaluated the toxicity of DEHP and B[a]P as a mixture (B[a]P + DEHP: 5 mg/kg + 300 mg/kg and 10 mg/kg and 600 mg/kg) given on alternate days for 60 days via oral gavage. The authors found that the mixture was toxic, as evidenced by the mixture decreasing 17β-estradiol levels, increasing estrous cycle duration, decreasing primordial, primary, and secondary follicle numbers, increasing atretic follicles, and decreasing *Cyp19a1* expression [37]. In another study, adult C57BL/6 J female mice were exposed orally to a mixture of phthalates (DEHP at 5 µg/kg/day, DBP at 0.5 µg/kg/day, BBP at 0.5 µg/kg/day, DiBP at 0.5 µg/kg/day, and DEP at 0.25 µg/kg/day) for six weeks. The results indicated that the mice exposed to the phthalate mixture had longer estrous cycles, with decreased time in proestrus and increased time in estrus compared to controls [38] (Table 1).

Recently, studies have started to compare the effects of exposure to single phthalates with the effects of exposure to mixtures of phthalates during adulthood on ovarian and female reproductive outcomes. It is likely that individual phthalates in the mixture, particularly DEHP, contributed to the toxicity of the mixture. In one study, Li et al. evaluated the prepuberal effects of intraperitoneal exposure to DEHP (0, 20, and 40 µg/kg at 5 dpp, 10 dpp, and 15 dpp) on antral follicle growth [39]. Phthalate-exposed ovaries collected at 20 dpp had a smaller volume compared to controls. In addition, DEHP exposure resulted in a decreased percentage of large antral follicles, upregulation of apoptosis-related genes, and inhibition of cell-proliferation-related genes compared to controls. DEHP exposure also resulted in reactive oxygen species accumulation and decreased expression of antioxidant enzymes, suggesting that DEHP exposure induces oxidative stress and apoptosis, impacting ovarian follicle growth during the prepuberal stage in mice [39]. In another study, the effects of DEHP were evaluated in lactating mice. DEHP (20 and 40 µg/kg) was orally administered every day to the dams until the nursing pups reached 21 dpp. DEHP exposure decreased the numbers of oocytes, primordial follicles, and antral follicles in the nursing pups compared to controls [40] (Table 1). Further, DEHP exposure altered steroidogenic regulators compared to controls, resulting in decreased levels of estradiol in both the lactating dams and the nursing pups, indicating that DEHP exposure dysregulates the steroidogenic process. Finally, DEHP exposure reduced granulosa cell proliferation and increased DNA damage and apoptosis compared to controls in the nursing pups. These data suggest that lactational exposure to DEHP can affect the secretion of hormones and the development of antral follicles in the pups, but also in the mother. In a study by Hannon et al. (2014), oral exposure to DEHP (20 µg/kg/day–750 mg/kg/day daily for 10 and 30 days) resulted in reproductive alterations in CD-1 mice. DEHP exposure prolonged estrous duration and accelerated primordial follicle recruitment [41] (Table 1). Similarly, in a study using SD rats to evaluate the toxicity of DEHP (300 mg/kg and 600 mg/kg), DEHP exposure decreased 17β-estradiol levels, prolonged the duration of the estrous cycle, decreased primary and secondary follicle numbers, and increased atretic follicles compared to controls [37] (Table 1). In another study, DEHP-exposed mice (5 µg/kg/day or 50 µg/kg/day) had longer estrous cycles, with decreased time in proestrus and increased time in estrus compared to controls [38] (Table 1).

Interestingly, phthalate exposure can have persistent or long-term effects on ovarian function. In a study by Hannon et al. (2016), oral exposure to DEHP (20 µg/kg/day–500 mg/kg/day) for 10 days resulted in altered estrous cyclicity by increasing the percentage of days mice spent in estrus and decreasing the percentage of days spent in metestrus/diestrus compared to controls at 6 months postdosing, and by decreasing the percentage of days spent in estrus and increasing the percentage of days spent in metestrus/diestrus at 9 months postdosing. DEHP exposure also decreased inhibin B levels, increased the BAX/BCL2 ratio in primordial follicles, and decreased primordial and total follicle numbers at 9 months postdosing compared to controls. Based on the results, the authors suggested a persistent effect of this phthalate, leading to reproductive aging [42]. The findings of Hannon et al. are consistent with a study by Chiang et al. (2020), in which the effects of 10 days of exposure to DEHP or DiNP (20 µmg/kg/day–200 mg/kg/day) were evaluated at 12, 15, and 18 months postdosing. DEHP and DiNP disrupted estrous cyclicity, increased pregnancy loss, decreased fertility, altered the sex ratio of pups, altered ovarian follicle populations, and disrupted hormone levels at 12–18 months postdosing, suggesting that short-term exposure to DEHP and DiNP during adulthood has long-term consequences in late life [43] (Table 1).

In addition, phthalates have been shown to have transgenerational effects on reproductive health. One study assessed the transgenerational effects of maternal DEHP exposure on folliculogenesis. In that study, pregnant mice were exposed to DEHP or a vehicle control from day 0.5 of gestation until weaning at postnatal day (PND) 21. The exposure window was selected to cover the critical time period of reproductive development in the mouse. DEHP was added to the chow at 0.05 mg/kg/day and 5 mg/kg/day. To examine the transmission effects of DEHP, mice in each treatment group were used to produce F1, F2, and F3 offspring. All offspring were collected at PND 42. The results indicated a significant decrease in the number of primordial follicles in all treatment groups in the F1–F3 generations compared to controls. Maternal exposure to DEHP also significantly increased the number of preantral follicles in all treatment groups throughout all generations compared to controls. Lastly, the data showed that the phthalate exposure significantly decreased the number of antral follicles in the F1 and F2 generation compared to controls [44].

DEHP has been widely researched and shown to have negative impacts on female reproduction; however, this is not the case for all phthalates present in mixture studies. In one study, immature female rats were dosed with 20 and 200 mg/kg of BBP and 10 and 100 mg/kg of DBP separately for three consecutive days from PND 21, and another group of rats was dosed with the same chemicals from PND 21 daily for 20 days [45]. The results of the 3-day study indicated a significant increase in body weight at the 10 and 20 mg/kg doses of both DBP and BBP compared to controls. Further, DBP and BBP at the 100 and 200 mg/kg doses significantly decreased uterine weight compared to controls. Additionally, in the 20-day study, both DBP and BBP significantly decreased body weight on PND 27, 33, and 42 compared to controls. Collectively, the data from the study suggest that DBP and BBP can decrease body and uterine weight, but may not have estrogenic potential in vivo [45].

Another phthalate present in mixture studies is DiBP. Unlike other phthalates, studies assessing the effects of DiBP on female reproductive outcomes are limited. DiBP was historically used less compared to other phthalates, and hence it has been studied less than other phthalates [46]. However, DiBP has been shown to affect maternal body weight gained during gestation or lactation, alter gestation length, and cause a change in morphological development by altering anogenital distance (AGD), displacing the ovaries, and changing the time to the onset of puberty. Other known effects of DiBP include a change in reproductive organ weights of the uterus, vagina, and ovary [46]. However, the majority of studies assessing DiBP exposure focus on its impact on male-related outcomes [46]. This highlights a major gap in research and the need for more studies addressing female reproductive outcomes, especially focusing on DiBP and other understudied phthalates common in human exposure. While studies examining the effects of phthalate mixtures on ovarian function and female reproduction exist, they are limited in number and scope. Due to the known adverse effects of some single phthalates on the ovary and female reproduction, it is imperative to look more closely at all chemicals that are environmentally relevant and mimic real-world scenarios of exposure. Furthermore, the currently limited amount of information on phthalate toxicity highlights a need for future studies examining the effects of exposure to mixtures during adulthood on adult reproductive outcomes. This will give insights into the mechanisms by which phthalate mixtures cause toxicity and how phthalate exposures may negatively impact future generations.

**Table 1:** Effects of single phthalates and phthalate mixtures on the ovary and female reproduction in vivo.

Reference	Exposure	Main Findings
Li et al., 2016 [39]	Mouse (CD-1) Prepubertal exposure from PND 5–15 to DEHP (0, 20, and 40 µg/kg/day) via intraperitoneal exposure every 5 days; tissues collected at PND 20	- Smaller ovarian volume (20 and 40 µg/kg/day) - Decreased percentage of large antral follicles (20 and 40 µg/kg/day) - Upregulation of apoptosis-related genes and inhibition of cell-proliferation-related genes (20 and 40 µg/kg/day)
Liu et al., 2021 [40]	Mouse (ICR) Lactating mice exposed to DEHP until nursing mice reached PND 21 (20 and 40 µg/kg/day) via oral dosing; tissues collected at PND 21	- Decreased number of oocytes, primordial follicles, and antral follicles in the nursing pups (20 and 40 µg/kg/day) - Altered steroidogenic regulators (20 and 40 µg/kg/day) - Decreased estradiol levels in the lactating dams and the nursing pups (20 and 40 µg/kg/day) - Reduced granulosa cell proliferation and increased DNA damage and apoptosis (20 and 40 µg/kg/day)
Hannon et al., 2014 [41]	Mouse (CD-1) Adult exposure to DEHP (20 µg/kg/day–750 mg/kg/day daily) for 10 or 30 days via oral dosing; tissues collected following the dosing period	10-day exposure: - Prolonged estrous duration (20 µg/kg/day, 20, 200, and 750 mg/kg/day) - Decreased primordial follicle number (20 and 200 mg/kg/day) - Increased primary follicle number (20 µg/kg/day, 200 µg/kg/day, and 750 mg/kg/day) - 30-day exposure: Prolonged estrous duration (200 mg/kg/day) - Increased primary follicle number (200 µg/kg/day and 200 mg/kg/day)
Hannon et al., 2016 [42]	Mouse (CD-1) Adult exposure to DEHP (20 µg/kg/day–500 mg/kg/day) for 10 days via oral dosing; tissues collected at 6 and 9 months	Six months postdosing: - Increased time spent in estrus (20 and 200 mg/kg/day) - Decreased time spent in metestrus/diestrus (20 mg/kg/day) Nine months postdosing: - Decreased time spent in estrus (500 mg/kg/day) - Increased time spent in metestrus/diestrus (500 mg/kg/day) - Increased progesterone levels (500 mg/kg/day) - Decreased primordial number (20, 200, and 500 mg/kg/day) - Decreased total follicle number (200 and 500 mg/kg/day)
Chiang et al., 2020 [43]	Mouse (CD-1) Adult exposure to DEHP (20 µg/kg/day–200 mg/kg/day) and DiNP (20 µg/kg/day–200 mg/kg/day) for 10 days via oral dosing; tissues collected at 12, 15, and 18 months	12 months postdosing: - Increased time spent in estrus (DEHP; 20 µg/kg/day) - Decreased primordial follicle number (DiNP; 20 µg/kg/day and 200 mg/kg/day) - Decreased primary follicle number (DEHP; 20 mg/kg/day) - Decreased gestational index (DEHP; 20 mg/kg/day) - Decreased percentage of female pups (DEHP; 20 mg/kg/day, DiNP; 20 µg/kg/day) - Decreased litter size (DEHP; 200 µg/kg/day) 15 months postdosing: - Increased time spent in estrus (DEHP; 20 µg/kg/day) - Increased preantral follicle number (DEHP; 200 µg/kg/day, DiNP; 100 µg/kg/day) - Decreased antral follicle number (DEHP; 200 µg/kg/day, 200 mg/kg/day) 18 months postdosing: - Increased primordial follicle number (DiNP; 100 µg/kg/day, 200 mg/kg/day) - Decreased antral follicle number (DiNP; 200 µg/kg/day) - Decreased testosterone levels (DiNP; 100 µg/kg/day) - Decreased estradiol levels (DiNP; 100 µg/kg/day)
Xu et al., 2010 [37]	Rat (SD) Young rat exposed to DEHP and B[a]P alone or as a mixture (B[a]P: 5 mg/kg/day and 10 mg/kg/day; DEHP: 300 mg/kg/day and 600 mg/kg/day; B[a]P + DEHP: 5 mg/kg/day + 300 mg/kg/day and 10 mg/kg/day and 600 mg/kg/day) on alternate days for 60 days via oral gavage; tissues collected at 60 days	- Decreased 17β-estradiol levels (DEHP; 300 mg/kg/day and 600 mg/kg/day, B[a]P + DEHP mixture; 5 mg/kg/day + 300 mg/kg/day and 10 mg/kg/day and 600 mg/kg/day - Increased estrous cycle (DEHP; 300 mg/kg/day and 600 mg/kg/day, and B[a]P + DEHP mixture; 5 mg/day + 300 mg/kg/day and 10 mg/kg/day and 600 mg/kg/day) - Decreased primordial follicle number (B[a]P + DEHP mixture; 10 mg/kg/day and 600 mg/kg/day) - Decreased primary/secondary follicle numbers (DEHP; 600 mg/kg/day, and B[a]P + DEHP mixture; 10 mg/kg/day and 600 mg/kg/day) - Increased atretic follicle number (DEHP 600 mg/kg/day, and B[a]P + DEHP mixture; 5 mg/kg + 300 mg/kg/day and 10 mg/kg/day and 600 mg/kg/day) - Decreased Cyp19a1 mRNA and CYP19A1 protein levels (DEHP; 300 mg/kg/day and 600 mg/kg/day, and B[a]P + DEHP mixture; 5 mg/kg+ 300 mg/kg/day and 10 mg/kg/day and 600 mg/kg/day)
Adam et al., 2021 [38]	Mouse (C57BL/6J) Adult exposure to DEHP (5 µg/kg/day and 50 µg/kg/day) or a mixture of phthalates for 6 weeks via oral dosing; tissues collected after week 7	- Increased estrous cycle duration (DEHP; 5 and 50 µg/kg/day, and mixture) - Decreased time spent in proestrus (DEHP; 5 and 50 µg/kg/day, and mixture) - Increased time spent in estrus (DEHP; 5 and 50 µg/kg/day, and mixture)
Ahmad et al., 2013 [45]	Rat (strain unknown) Young rats exposed to BBP (20 and 200 mg/kg/day) or DiBP (10 and 100 mg/kg/day) for three consecutive days via oral dosing; tissues collected on day 4 Study 2: Young rats exposed to BBP (20 and 200 mg/kg/day) or DiBP (10 and 100 mg/kg/day) from PND 21 for 20 days; tissues collected on PND 42	- Increased body weight (BBP; 20 mg/kg/day, and DBP; 10 mg/kg/day) - Decreased uterine weight (BBP; 200 mg/kg/day, and DBP; 100 mg/kg/day) - Decreased body weight (BBP; 20 mg/kg/day, 200 mg/kg/day, and DBP; 10 mg/kg/day, 100 mg/kg/day) at PND 27, 33, and 42
Pocar et al., 2017 [44]	Mouse (CD-1) Prenatal exposure to DEHP (0.05 mg/kg/day, 5 mg/kg/day) from GD 0.5–PND 21 via chow; pups examined at PND 21, F1–F3 generation tissues collected at PND 42	- Decreased number of primordial follicles (DEHP; 0.05 mg/kg/day, 5 mg/kg/day) in the F1–F3 generations - Increased number of preantral follicles (DEHP; 0.05 mg/kg/day, 5 mg/kg/day) in the F1–F3 generations - Decreased number of antral follicles in the F1 and F2 generations (DEHP; 0.05 mg/kg/day) - Decreased number of antral follicles (DEHP; 5 mg/kg/day) in the F1 generation

## 8. Effects of Prenatal Single and Phthalate Mixture Exposure on the Ovary and Female Reproduction in Offspring

In addition to examining the effects of postnatal exposure to phthalate mixtures on adult ovarian and reproductive outcomes, it is important to determine the consequences of prenatal exposure to phthalate mixtures on the ovarian and reproductive health of the female offspring. This is because several studies show that the prenatal window of exposure is a sensitive time period and that exposure to EDCs such as phthalates in this time period can have long-term effects on the offspring [13]. Studies have also shown that phthalates can cross placental barriers and affect the developing fetus during pregnancy [15]. Currently, some studies have assessed the effects of prenatal exposure to phthalate mixtures in F1 female mice, but the research is limited to very few mixtures. A series of studies conducted in mice focus on a phthalate mixture composed of 35% DEP, 21% DEHP, 15% DBP, 15% DiNP, 8% DiBP, and 5% BBzP. It is important to note that this mixture was derived from levels of phthalate metabolites found in the urine samples of pregnant women in an epidemiological study called the IKIDS study [47]. The studies also used doses of the mixture that are relevant to human exposure levels. In humans, adult daily exposure to DEP ranges from 2.32 to 12  µg/kg/day, DEHP exposure ranges from 3 to 30  µg/kg/day, DBP exposure ranges from 0.84 to 5.22  µg/kg/day, DiBP exposure ranges from 0.12 to 1.4  µg/kg/day, and BBP exposure ranges from 0.26 to 0.88  µg/kg/day [14,48]. One study using this specific phthalate mixture orally dosed pregnant dams with vehicle control (oil) or the phthalate mixture at 20 μg/kg/day–500 mg/kg/day. The dams gave birth naturally, and the sera and ovaries from F1 female offspring were collected on postnatal day 60. The results showed that the phthalate mixture disrupted folliculogenesis by causing a decrease in the amount of FSH, an increase in the percentage of primordial follicles, a decrease in the percentage of preantral and antral follicles, and a decrease in the number of preantral follicles compared to controls (Table 2) [47]. Lastly, the phthalate mixture disrupted steroidogenesis by significantly decreasing the amount of estradiol, testosterone, and progesterone in the F1 females compared to controls. It is likely that the phthalate mixture decreased prenatal hormone levels by interfering with steroidogenic regulators. The phthalate mixture caused a decrease in the steroidogenic regulators *Star*, *Cyp11a1*, *Cyp17a1*, and *Cyp19a1* [47] (Table 2).

Another study used the same phthalate mixture and examined its effects on other reproductive outcomes in F1 offspring at postnatal days 8 and 60, and at 3 months and 6 months. The results showed that the phthalate mixture disrupted estrous cyclicity, reduced fertility, increased uterine weight, decreased anogenital distance, induced cystic ovaries, and caused breeding problems in the F1 females [49] (Table 2).

Another study assessing phthalate mixtures on female reproductive health used the same phthalate mixture and showed that prenatal exposure to the mixture could accelerate the natural age-related decline in reproductive function by decreasing the time spent in proestrus and decreasing the ability of the F1 generation to carry out pregnancy and produce pups at 11 and 13 months [50].

Additionally, a separate study used a phthalate mixture composed of the five phthalate esters BBP, DBP, DEHP, DiBP, and DPeP in a ratio of 3:3:3:3:1. This ratio was used so that each phthalate contributed equally to the reduction in testicular testosterone production in fetal male rats, but outcomes were examined in both males and females. The study was composed of two individual studies. Pregnant dams were dosed via oral gavage with the mixture at 0, 65, 130, 260, 520, and 780 mg /kg/day from gestational day gestational day (GD) 8 to PND 3. Part two of this study consisted of oral gavage dosing with the same mixture at 520 mg/kg/day for one of three dosing periods: GD 8–19 (continuous), GD 8–13 (early), or GD 14–19 (late), with a control group dosed with corn oil from GD 8–19. Both studies allowed pups to be delivered naturally and recorded body weight and anogenital distance of pups at PND 2. The first study collected offspring at PND 77 and PND 350, whereas the second study collected offspring at PND 120. The results showed a significant increase in fetal mortality and a decrease in the total number of fetuses at 780 mg/kg/day compared to the control group. On PND 2, the 780 mg/kg/day treatment significantly decreased the body weight of female pups compared to the control group. The results also showed a significant increase in the absence of a vaginal opening at 520 and 780 mg/kg/day. Further, continuous dosing from GD 8–19 and GD 8–13 led to an increase in the absence of a vaginal opening in the offspring compared to controls. Female anogenital distance was not affected by any dosing period [51].

Lastly, one study used a phthalate mixture composed of four phthalate monoesters: 33% MBP, 16% MBzP, 21% MEHP, and 30% MNP. This mixture was derived from a study that identified 20 phthalates suspected to be EDCs and contained phthalates that are commonly present in the first-trimester urine/serum of pregnant women [52]. The serum and urine levels of phthalates were then used to estimate the daily intake of their active monoesters [52]. In the study, pregnant mice were exposed to 0, 0.26, 2.6, and 13 mg/kg/day of the phthalate mixture (representing 0×, 10×, 100×, and 500× of the geometric mean of pregnant women’s serum levels for each chemical found in the mixture) from GD 0.5 until delivery. For comparison purposes, a control group was given DMSO. All animals in the study were dosed via food. The outcomes analyzed were the AGD index, gonadal histology, gene expression, and hormone levels of both male and female offspring. On PND 1, 21, and 90, AGD values were measured, and tissues from the animals were collected to examine prepubertal and adulthood effects of the mixture at PND 20 and 90. For the purposes of this review, only female outcomes will be addressed. The results indicated that at 0.26 mg/kg/day, the mixture significantly increased the AGD index on PND 1 compared to controls. On PND 21 the mixture significantly decreased the AGD at 0.26 mg/kg/day and 2.6 mg/kg/day compared to controls. However, the mixture only significantly decreased body weight at 2.6 mg/kg/day on PND 21 and PND 90 compared to controls. Data also showed that the mixture at all doses significantly reduced the number of secondary follicles and increased the number of atretic follicles compared to controls on PND 21.

Further, on PND 90, the mixture reduced levels of secondary follicles in all treatment groups and reduced primary follicles and increased atresia at 2.6 mm/kg/day and 13 mg/kg/day compared to controls. Gene expression data showed a decrease at 0.26 mg/kg/day and 2.6 mg/kg/day in the expression of *Cyp19a1* on PND 21 and decreased expression of *Cyp17a1* and *Star* at 13 mg/kg/day on PND 90. The expression of *Cyp17a1* was also decreased at 2.6 mg/kg/day on PND 90. Lastly, a separate experiment was conducted in vitro as part of the same study to determine whether steroidogenesis was directly or indirectly affecting follicle numbers. The human adrenocortical carcinoma cell line H295R, a validated cell line for studying steroidogenesis, was used, seeded in 96-well plates in triplicates, incubated overnight, and treated with 0.1×, 1×, 10×, 100×, and 1000× concentrations of the mixture or controls consisting of DMSO (0.1%), forskolin (10 µM), prochloraz (3 µM), and a non-treated control. All doses were analyzed after 48 h of exposure to the mixture. The mixture at 1× significantly decreased *Star* and *Hsd3b2* expression compared to the controls [53].

**Table 2:** Effects of prenatal exposure to phthalate mixtures on female reproduction.

Reference	Exposure	Main Findings
Zhou et al., 2017 [47]	Mouse (CD-1) Prenatal exposure from GD 10 to birth with a mixture of DEP, DEHP, DBP, DiBP, DiNP, and BzBP (20 μg/kg/day, 200 μg/kg/day, 200 mg/kg/day, and 500 mg/kg/day) via oral dosing of pregnant dams; tissues collected at PND 60	- Increased primordial follicle percentage (20 μg/kg/day) - Decreased preantral follicle number (20 μg/kg/day) - Decreased preantral (20 μg/kg/day, 200 mg/kg) and antral (200 μg/kg/day) follicle percentage - Increased atretic follicles (500 mg/kg/day) and atretic follicle percentage (200, 500 mg/kg/day) - Decreased FSH (500 mg/kg/day) and estradiol (20 μg/kg/day, 200 mg/kg/day, 500 mg/kg/day) - Decreased progesterone (500 mg/kg/day) - Decreased testosterone (200 μg/kg/day, 200 mg/kg/day, 500 mg/kg/day) - Decreased Cyp11a1 expression (20 μg/kg/day) - Decreased Star expression (20 μg/kg/day, 200 mg/kg/day, 500 mg/kg/day) - Decreased aromatase expression (200 μg/kg/day)
Gill et al., 2021 [49]	Mouse (CD-1) Prenatal exposure from GD 10 to birth with a mixture of DEP, DEHP, DBP, DiBP, DiNP, and BzBP (20 μg/kg/day, 200 μg/kg/day, 200 mg/kg/day, and 500 mg/kg/day) via oral dosing of pregnant dams; tissues collected at PND 8 and 60, and at 3 months and 6 months	- Decreased anogenital distance (500 mg/kg/day) at PND 8 - Increased uterine weight (20 μg/kg/day) at PND 8 - Decreased anogenital distance (20, 200 μg/kg/day) PND 60 - Increased uterine weight (500 mg/kg/day) at PND 60 - Decreased body weights at vaginal opening (20 μg/kg/day) - Reduced days between vaginal opening and first estrus (200 μg/kg/day) - Increased time spent in estrus (20 and 200 μg/kg/day, 200 and 500 mg/kg/day) at 3 months - Decreased time in metestrus and diestrus (20 and 200 μg/kg/day, 200 and 500 mg/kg/day) at 3 months - Decreased time spent in proestrus (20 and 200 μg/kg/day, 200 mg/kg/day) at 6 months - Increased time spent in estrus (500 mg/kg/day) at 6 monthsIncreased number of days needed to become pregnant (200 mg/kg/day) at 3 monthsReduced fertility and pregnancy rate (200 and 500 mg/kg/day) at 3 monthsDecreased number of live pups born (200 μg/kg/day) at 6 months
Brehm et al., 2021 [50]	Mouse (CD1) Prenatal exposure from GD 10 to birth with a mixture of DEP, DEHP, DBP, DiBP, DiNP, and BzBP (20 μg/kg/day, 200 μg/kg/day, 200 mg/kg/day via oral dosing of pregnant dams; tissues collected at 11 and 13 months	F1 generation: - Decreased time spent in proestrus (200 μg/kg/day) at 11 and 13 months - Decreased amount of time spent in proestrus (20 μg/kg/day) at 13 months - Decreased in percentage of females that gave birth at 11 and 13 months - Decreased time to pregnancy and percentage of F1 females who gave birth (200 μg/kg/day) at 13 months F2 generation: - Decreased % female pups (20 and 200 μg/kg/day) - Decreased female pups (20 μg/kg/day) at 13 months
Hannas et al., 2013 [51]	Rat (SD) Prenatal exposure from GD 8–PND 3. to a mix of BBP, DBP, DEHP, DiBP, and DPeP (0, 65, 130, 260, 520, and 780 mg/kg/day) via oral gavage; pups examined at PND 2, tissues collected at PND 77 and PND 350 Part 2, Rat (SD) Prenatal exposure from GD 8–19, GD 8–13, or GD 14–19 to the mixture (520 mg/kg/day) via oral gavage dosing; tissues collected at PND 120	Increased fetal mortality (780 mg/kg/day) - Decreased total number of fetuses (780 mg/kg/day) - Decreased body weight of female pups (780 mg/kg/day) at PND 2 - No change in anogenital distance Part 2 - Increased absence of vaginal opening (520 and 780 mg/kg/day) at GD 8–19
Repouskou et al., 2019 [53]	Mouse (C57/BL6) Gestational exposure from GD 0.5 until birth to a mixture of MBP, MBzP, MEHP, and MNP (0 mg/kg/day, 0.26 mg/kg/day, 2.6 mg/kg/day, and 13 mg/kg/day) via food of pregnant dams; examined at PND 1; tissues collected at PND 21 and PND 90	- Increased anogenital index (0.26 mg/kg/day) at PND 1 - Decreased anogenital index (0.26 mg/kg/day and 2.6 mg/kg) at PND 21 - Decreased body weight (2.6 mg/kg/day) at PND 21 and PND 90 - Decreased number of secondary follicles (0.26 mg/kg/day, 2.6 mg/kg/day, and 13 mg/kg/day) at PND 21 - Increased number of atretic follicles (0.26 mg/kg/day, 2.6 mg/kg/day, and 13 mg/kg/day) at PND 21 - Reduced levels of secondary follicles (0.26 mg/kg/day, 2.6 mg/kg/day, and 13 mg/kg/day) at PND 90 - Reduced primary follicles (2.6 mg/kg/day and 13 mg/kg/day) at PND 90 - Increased levels of atretic follicles (2.6 mg/kg/day and 13 mg/kg/day) at PND 90 - Decrease in Cyp19a1 (0.26 mg/kg/day and 2.6 mg/kg/day) at PND 21 - Decrease in Star (13 mg/kg/day) at PND 90 - Decrease in Cyp17a1 (2.6 mg/kg/day and 13 mg/kg/day) at PND 90

Finally, a few studies have examined whether prenatal exposure to a phthalate mixture (35% DEP, 21% DEHP, 15% DBP, 15% DiNP, 8% DiBP, and 5% BBzP) has multigenerational and transgenerational effects on female reproduction. The results of the studies showed that the phthalate mixture decreased the percentage of antral follicles and testosterone levels in the F2 generation. Further, the mixture caused a change in follicle number and a decrease in the levels of LH compared to controls in the F3 generation [54]. The phthalate mixture also caused other reproductive effects such as increased uterine weight, decreased anogenital distance, and fertility complications in the F2 generation. It also increased uterine and ovarian weight, increased the metestrus/diestrus phase, decreased anogenital distance, and increased fertility complications in the F3 generation [47,54].

The mixture used in several previous studies was environmentally relevant and mimicked a real-world scenario; however, humans throughout the world are frequently exposed to other mixtures. Further, the published phthalate mixture studies all examine different compounds of phthalates, two focusing on phthalate diesters and the other focusing on phthalate monoesters. In the two phthalate diester studies, some of the same phthalates were used, but different doses were examined [47,49,50,51]. In the phthalate monoester study, some monoesters of the phthalates were used, but not all potential monoesters were studied [53]. All studies indicate that phthalates may have some negative impacts on female reproductive outcomes, but they are all very different in their approach. The studies highlight the need for more research, but cannot be closely compared due to their broad differences in phthalate compounds and doses [55,56,57,58].

Although studying phthalate mixtures mimics human exposure to phthalates, studies on the effects of prenatal exposure to single phthalates are useful for predicting which phthalate within a mixture may be contributing to a given effect. Numerous rodent studies indicate that prenatal exposure to a single phthalate such as DEHP can have deleterious effects on the reproductive health and fertility of female offspring [55,57,59,60,61,62,63]. These studies demonstrate that the ovary is sensitive to the toxic effects of prenatal exposure to DEHP. Interestingly, the effects of acute phthalate exposure during this critical window of development can be detected throughout the life of female offspring. Specifically, mice prenatally exposed to DEHP had decreased total follicle numbers and increased follicle atresia compared to controls, suggesting that follicles at all stages of maturity are susceptible to phthalate-induced toxicity (Table 3) [55,61]. Further, mice prenatally exposed to DEHP had decreased primordial and antral follicles and increased primary and preantral follicle numbers compared to controls, indicating that DEHP exposure may accelerate folliculogenesis in mice [56,57,58,64].

Steroid hormone production is also impaired with prenatal exposure to single phthalates (Table 3). Single phthalates (in this case DEHP) have variable effects on serum gonadotropin levels in rodents. Prenatal exposure to DEHP increased LH and decreased FSH and inhibin β levels during diestrus compared to controls in mice [55]. In contrast, prenatal exposure to DEHP increased FSH during proestrus compared to controls in rats [59]. Prenatal exposure to MEHP, a DEHP metabolite, increased FSH compared to controls in mice briefly exposed during gestational days 17–19 [65]. Prenatal DEHP exposure also disrupted ovarian steroidogenesis by increasing estradiol and decreasing testosterone in diestrus compared to controls in mice [55,56]. Further, prenatal DEHP exposure has been shown to decrease estradiol levels in proestrus compared to controls in rats [59]. Prenatal exposure to MEHP also increased estradiol levels in mice exposed from GD 17 to 19 [65]. These changes in hormone levels probably result from altered expression of steroidogenic enzymes. DEHP and MEHP exposure decreased the expression of *Star*, *Cyp11a1*, *Cyp17a1*, and *Cyp19a1*, whereas DEHP exposure increased the expression of *Hsd3b1* [57,61,63,65]. DEHP-exposed mice may also experience disruptions in hormone signaling due to altered expression of hormone receptors. Prenatal DEHP exposure increased the expression of estrogen receptor 2 (*Esr2*) and decreased the expression of *Esr1*, FSH receptor (*Fshr*), LH receptor (*Lhr*), and androgen receptor (*Ar*) compared to controls [57,63].

Prenatal exposure to single phthalates has been associated with adverse fertility outcomes in mice. Mice prenatally exposed to DEHP exhibited earlier age at first estrus, whereas mice prenatally exposed to MEHP exhibited a delayed onset of first estrus compared to controls [60,65]. Phthalate-induced changes in estrous cyclicity have also been reported in mice. Mice prenatally exposed to DEHP spent less time in estrus and more time in proestrus, metestrus, and diestrus than controls [55,60]. In contrast, mice prenatally exposed to the DEHP metabolite MEHP spent more time in estrus and metestrus and less time in proestrus and diestrus than controls [65]. Prenatally DEHP-exposed mice exhibited a lower fertility index, a measure of the number of pregnant females relative to females with copulatory vaginal sperm plugs [60].

In addition to having persistent effects in F1 female offspring, prenatal single phthalate exposure exhibits multigenerational and transgenerational effects (Table 3). Exposing pregnant dams to DEHP led to decreased methylation of maternally imprinted genes in primordial germ cells and oocytes from F2 offspring, decreased global DNA methylation in ovaries from F3 offspring, and decreased expression of methylation-related factors in ovaries isolated from the F2 and F3 generations [62,63]. Additionally, DEHP exposure altered folliculogenesis in a multigenerational and transgenerational manner. In ovaries from F2 and F3 offspring at PND 21 exposed ancestrally to DEHP, the follicle populations shifted, resulting in decreased populations of immature follicles and increased populations of mature follicles compared to controls [56,57]. Interestingly, at 12 months of age, ancestral DEHP exposure, particularly in the F3 generation, was associated with an increased percentage of primordial follicles and decreased percentage of primary follicles compared to controls [55]. It is evident that ancestral exposure to DEHP alters folliculogenesis across the lifetime of the exposed animal, perhaps through accelerating follicle maturation and depletion early in life and impairing follicle development later in life.

Furthermore, DEHP exposure disrupts steroidogenesis by decreasing progesterone and testosterone production in the F2 generation and increasing estradiol and FSH production and decreasing testosterone production in the F3 generation compared to controls [55,56]. These changes in hormone production are associated with altered expression of steroidogenic enzymes, including decreased expression of *Hsd17b1* and *Cyp19a1* [63]. These changes in folliculogenesis and steroidogenesis in the F2 and F3 generations following DEHP exposure culminate in the disruption of reproductive parameters. Mice ancestrally exposed to DEHP exhibit vaginal opening at an earlier age (F3 generation) and first estrus is detected at an earlier age (F2 generation), suggesting accelerated onset of puberty in exposed mice compared to unexposed mice [60]. Exposure to DEHP caused shifts in estrous cyclicity, including increased time spent in estrus in F2 and F3 mice [60]. Further, F2 females exposed ancestrally to DEHP exhibited a lower gestational index, a measure of females who successfully delivered pups relative to the total number of pregnant females, suggesting fertility is compromised following ancestral DEHP exposure [60].

In summary, all phthalate studies focusing on prenatal exposure to mixtures or single phthalates show that phthalates have the potential to cause negative reproductive outcomes in female offspring. However, major differences in these studies were the chemicals used to compose the mixture and the doses used in the experiments. While each study provides insight into how phthalates can impact female reproduction, they are very different in their approach. Understanding the effects of prenatal exposure to environmentally relevant phthalates is important for determining the long-term consequences of phthalate exposure on the ovary and female reproduction. Females can be exposed to phthalates throughout their entire reproductive lifespan. This means that every stage of development within the ovary is targeted, as well as every generation. Understanding the impact on the offspring helps determine whether phthalates have permanent reproductive effects on the ovary or whether there is a possible compensation over time to alleviate the negative effects in future generations. More studies evaluating prenatal exposure to phthalates are needed to answer persisting questions regarding the long-term effects of phthalate mixtures as well as single phthalates on the ovary and female reproductive health.

## 9. Conclusions

**Figure 2:**
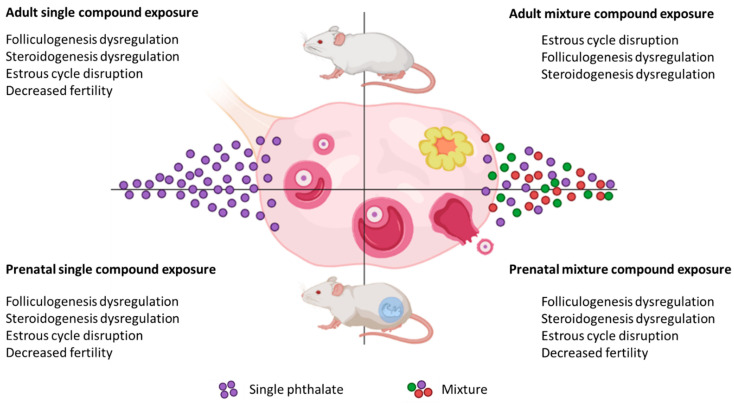
Effects of phthalates on female reproduction. The schematic shows that exposure to single phthalates as well as mixtures of phthalates affects several similar ovarian and female reproductive outcomes in mice.

The continual structural changes that the ovaries undergo at critical time points of development within a female’s lifespan and reproductive cycle make the ovaries a susceptible target for phthalates to disrupt normal reproductive function. Current data suggest that phthalates affect reproductive health by targeting the stages of folliculogenesis and steroidogenesis, while also impacting other reproductive outcomes (Figure 2).

In summary, phthalates are EDCs that can have different effects at different doses. EDCs often do not show a monotonic dose–response relationship and can show effects at low levels [66]. Although studies have assessed the effects of single phthalates and phthalate mixtures on ovarian and female reproductive health, limited information is available on multiple environmentally relevant phthalates and their long-term effects on ovarian and reproductive function. Therefore, single phthalates, especially those that are environmentally relevant and present in mixtures, should be more closely evaluated, to help determine whether these compounds behave synergistically or independently to cause reproductive toxicity. Some phthalates are more frequently used than others but should be compared with other phthalates to which humans are exposed on a regular basis. Without proper research and independently repeated studies addressing the impacts of phthalates on female reproduction, a degree of uncertainty occurs in concluding how all phthalates behave and which are causing the most negative outcomes. It is also important to design studies that focus on female reproductive outcomes and to evaluate the impacts of single phthalates and phthalate mixtures, due to differences in development and windows of susceptibility to EDCs. More studies focused on examining different endpoints will ensure that the information provided is consistent and supported by multiple studies. By better understanding the impact of both prenatal and postnatal exposure to phthalates and phthalate mixtures on the ovary in vitro and in vivo, we will be able to develop better strategies to prevent or treat phthalate-induced toxicity.

## Figures and Tables

**Table 3:** Effects of prenatal exposure to single phthalates on female reproduction in vivo.

Reference	Exposure	Main Findings
Rattan et al., 2019 [63]	Mouse (CD-1) Prenatal exposure from GD 10.5 to birth to DEHP (20 μg/kg/day, 200 μg/kg/day, 500 mg/kg/day, or 750 mg/kg/day) via oral dosing of pregnant dam; F1–F3 generations euthanized at PND 21, and ovaries collected	F1 generation: - Increased Esr2 (750 mg/kg/day) and decreased Ar (20 μg/kg/day) expression - Increased Hsd3b1 (750 mg/kg/day) expression
Mirihagalle et al., 2019 [64]	Mouse (CD-1) Prenatal exposure from GD 10.5 to birth to DEHP (20 μg/kg/day DEHP + control diet or 20 μg/kg/day DEHP + high-fat diet) via diet of pregnant dam; estrous cyclicity and fertility monitored at 3, 6, and 9 months in F1–F3, tissues collected at PND 21	- Increased preantral follicle populations at PND 21 (20 μg/kg/day DEHP + high-fat diet) at PND 21
Brehm et al., 2018 [55]	Mouse (CD-1) Prenatal exposure from GD 11 to birth to DEHP (20 µg/kg/day, 200 µg/kg/day, 500 mg/kg/day, and 750 mg/kg/day) via oral dosing of pregnant dam; ovaries collected from the F1–F3 generation at 12 months of age	F1 generation: - Altered estrous cyclicity (750 mg/kg/day) - Increased ovarian cysts (750 mg/kg/day) - Decreased total follicle number (750 mg/kg/day) - Increased estradiol (500 and 750 mg/kg/day) - Decreased testosterone (500 mg/kg/day) - Decreased FSH (500 mg/kg/day) - Increased LH (20 μg/kg/day) F2 generation: - Decreased AGD (200 μg/kg/day) - Altered follicle number (200 μg/kg/day and 500 mg/kg/day) - Decreased testosterone (20 μg/kg/day) - Decreased progesterone (200 μg/kg/day) F3 generation: - Altered estrous cyclicity (20 and 200 μg/kg/day, 500 and 750 mg/kg /day) - Decreased folliculogenesis (200 μg/kg/day, 500 mg/kg/day) - Increased estradiol (20 μg/kg/day) - Decreased testosterone (20 μg/kg/day, 500 mg/kg/day) - Increased FSH (500 mg/kg/day)
Rattan et al., 2018 [56]	Mouse (CD-1) Prenatal exposure from GD 10.5 to birth to DEHP (20 μg/kg/day, 200 μg/kg/day, 200 mg/kg/day, 500 mg/kg/day, 750 mg/kg/day) via oral dosing of pregnant dams; F1–F3 generations euthanized at PND 21, and ovaries collected at PND 1, 8, 21, and 60, and at 3, 6, and 9 months	F1 generation: - Decreased ovarian weight at PND 21 (20 μg/kg/day, 750 mg/kg/day) - Increased ovarian weight at PND 21 (200 μg/kg/day) - Increased primary (20 μg/kg/day) and decreased antral (750 mg/kg/day) follicle percentages at PND 21 - Decreased atretic follicles (200, 500, and 750 mg/kg/day) at PND 60 - Increased estradiol (20 μg/kg/day, 750 mg/kg/day) at PND 8 and (500 mg/kg/day) at PND 60 - Decreased testosterone (200 μg/kg/day) at PND 60 F2 generation: - Decreased ovarian weight at PND 60 (200 μg/kg/day) - Increased preantral follicle percentage (200 mg/kg/day) at PND 8 - Decreased primordial (200 mg/kg/day) and antral follicle percentages (20 μg/kg/day) and increased preantral (200 mg/kg) and antral (200 mg/kg/day) follicle percentages at PND 21 - Decreased antral follicle percentage (200 μg/kg/day) at PND 60 - Increased progesterone at PND 21 (500 mg/kg/day) and decreased progesterone at PND 60 (500 and 750 mg/kg/day) F3 generation: - Decreased germ cell (500 and 750 mg/kg/day) and increased primordial follicle (500 and 750 mg/kg/day) percentages at PND 1 - Increased preantral follicle (20 μg/kg/day) percentage at PND 8
Rattan et al., 2018 [60]	Mouse (CD-1) Prenatal exposure from GD 10.5 to birth to DEHP (20 μg/kg/day, 200 μg/kg/day, 200 mg/kg/day, 500 mg/kg/day, 750 mg/kg/day) via oral dosing of pregnant dams; estrous cyclicity and fertility monitored at 3, 6, and 9 months in F1–F3	F1 generation: - Early age at first estrus (200 μg/kg/day) - Decreased time spent in estrus (200 μg/kg/day) at 9 months - Decreased fertility index (200 μg/kg/day) F2 generation: - Early age at first estrus (200 μg/kg/day) - Increased time spent in estrus (20 μg/kg/day) and decreased time spent in diestrus (200 μg/kg/day) at 9 months - Decreased gestational index (500 mg/kg/day) F3 generation: - Early age at vaginal opening (20 μg/kg/day, 500 mg/kg/day, and 750 mg/kg/day) - Increased time spent in estrus (20 μg/kg/day) and decreased time spent in diestrus (20 μg/kg/day) at 6 months
Wang et al., 2016 [61]	Mouse (ICR) Prenatal exposure from GD 0.5 to birth to DEHP (0, 0.02, 0.2, 2, 20, or 200 mg/kg/day) via oral dosing of pregnant dams; ovaries from F1 collected at PND 1 and 2	- Increased follicle atresia (2, 20, and 200 mg/kg/day) at PND 21 - Decreased Star expression at PND 1 (2, 20, and 200 mg/kg/day) and PND 21 (20 and 200 mg/kg/day) - Decreased Cyp11a1 expression at PND 1 (2, 20, and 200 mg/kg/day) and PND 21 (200 mg/kg/day)
Zhang et al., 2015 [57]	Mouse (CD-1) Prenatal exposure from 0.5 to 18.5 dpc to DEHP (DEHP 40 µg/kg/day) via addition to drinking water of pregnant dams; tissues collected at PND 21	- Decreased expression of Cyp17a1 and Cyp19a1 (40 µg/kg/day) in fetal F1 ovaries - Decreased primordial and increased secondary follicle populations at PND 21 (40 µg/kg/day) in the F1 generation - Decreased primordial and increased secondary follicle populations at PND 21 (40 µg/kg/day) in the F2 generation
Niermann et al., 2015 [58]	Mouse (CD-1) Prenatal exposure from GD 11 to birth to DEHP (20 μg/kg/day, 200 μg/kg/day, 200 mg/kg/day, 500 mg/kg/day, or 750 mg/kg/day) via oral dosing of pregnant dams; ovaries collected PND 1, 8, 21, and 60	- Decreased ovarian weight at PND 21 (20 μg/kg/day) - Increased uterine weight at PND 21 (200 μg/kg/day) - Increased preantral follicle counts at PND 21 (200 μg/kg/day, 500 mg/kg/day)
Meltzer et al., 2015 [59]	Rat (SD) Prenatal exposure from GD 14 to birth to DEHP (1, 20, 50, or 300 mg of DEHP/kg/day) via gavage of pregnant dams; ovaries collected at PND 60–68 in each stage of estrus	- Decreased estradiol levels at PND 60 (300 mg/kg/day) in proestrus (disrupted preovulatory estrogen surge) - Increased serum FSH in proestrus at PND 60 (1, 50, and 300 mg/kg/day) and estrus (50 and 300 mg/kg/day)
Moyer., 2012 [65]	Mouse (C57/BL6) Prenatal exposure from GD 17–19 to MEHP (100, 500, or 1000 mg/kg) via oral dosing of pregnant dams; collected at PND 56	- Delayed onset of first estrous cycle (500 and 1000 mg/kg/day) - Increased time spent in estrus (500 and 1000 mg/kg/day) and metestrus (100, 500, and 1000 mg/kg/day); decreased time spent in diestrus (500 and 1000 mg/kg/day) and proestrus (500 and 1000 mg/kg/day) in the PND 40–PND 56 age range - Early age when final litter delivered (275.5 days of age vs. 310.5 days of age) (1000 mg/kg/day) - Increased primary (500 and 1000 mg/kg/day), secondary (100, 500, and 1000 mg/kg/day), and antral (500 and 1000 mg/kg/day) follicle populations at PND 56 - Increased serum estradiol (1000 mg/kg/day) at PND 56 - Increased FSH (1000 mg/kg/day) and decreased estradiol (1000 mg/kg/day) at PND 365 - Decreased aromatase (500 and 1000 mg/kg/day) and Star (100, 500, and 1000 mg/kg/day) expression at PND 56

## Data Availability

Not applicable.
