# Correction: Fletcher et al. Effects of Phthalate Mixtures on Ovarian Folliculogenesis and Steroidogenesis. *Toxics* 2022, *10*, 251

**DOI:** 10.3390/toxics10100562

**Published:** 2022-09-26

**Authors:** Endia J. Fletcher, Ramsés Santacruz-Márquez, Vasiliki E. Mourikes, Alison M. Neff, Mary J. Laws, Jodi A. Flaws

**Affiliations:** Department of Comparative Biosciences, University of Illinois at Urbana-Champaign, Urbana, IL 61802, USA

The authors wish to make the following corrections to this paper [1]:

In the original publication, there was a mistake in Figure 1 as published. A label indicating “meiosis II” was used in the figure between the primary and secondary/preantral follicles. However, the label should have indicated that “meiosis II begins and is completed at fertilization”. The corrected Figure 1 appears below. The authors state that the scientific conclusions are unaffected. This correction was approved by the Academic Editor. The original publication has also been updated.

## Figures and Tables

**Figure 1:**
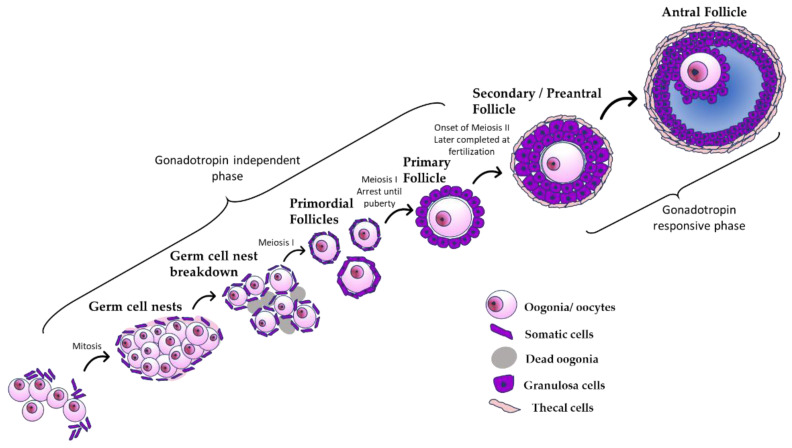
The process of folliculogenesis. The schematic shows that primordial germ cells form germ cell nests, which subsequently break down to form a finite pool of primordial follicles, starting the process of folliculogenesis. During folliculogenesis, primordial follicles grow and mature into primary follicles, then preantral follicles, and finally antral follicles.
